# The Bioenergy Potential of Date Palm Branch/Waste Through Reaction Modeling, Thermokinetic Data, Machine Learning KNN Analysis, and Techno-Economic Assessments (TEA)

**DOI:** 10.3390/polym17233182

**Published:** 2025-11-29

**Authors:** Abdulrazak Jinadu Otaru, Zaid Abdulhamid Alhulaybi Albin Zaid, Mubarak Mohammed Alkhaldi, Saud Mahmood Alholiby Albin Zaid, Abdullah AlShuaibi

**Affiliations:** 1 Department of Chemical Engineering, College of Engineering, King Faisal University, Al Ahsa 31982, Saudi Arabia; 2 College of Law, King Faisal University, Al Ahsa 31982, Saudi Arabia; 3 Materials Science & Engineering, Cornell University, Ithaca, NY 14850, USA

**Keywords:** date palm branch, pyrolysis, reaction modeling, KNN modeling, legal framework

## Abstract

This research assesses the bioenergy potential of date palm branch (DPB) waste, aligning with Saudi Arabia’s Vision 2030 energy and environmental goals. The study uses reaction modeling, thermokinetics, a k-nearest neighbors (KNN) machine learning approach, and techno-economic assessments. Experimental characterizations employing FTIR, SEM, and both proximate and ultimate analysis of pulverized DPB biomass reveal its lignocellulosic nature and compositional characteristics. Thermogravimetric analysis (TGA) of the material, tested between 25 and 1000 °C at heating rates of 7.5 to 60 °C per minute, revealed that the main thermal breakdown occurred from 200 to 530 °C, and was caused by the decomposition of hemicellulose and cellulose. Criado master plot analysis of the material’s thermal decomposition indicated the R3 contracting cylinder model was the most suitable reaction mechanism. The Jander [D3] and Ginstling–Brounshtein [D4] diffusion models were also good fits. The kinetic analysis showed that various model-free approaches, including FWO, KAS, STK, and FR, yielded comparable activation energy values for the hemicellulose and cellulose components, with the results clustering between approximately 98.43 and 109.30 kJ/mol. The application of the KNN machine learning technique in this study yielded accurate predictions ($R^{2}\sim 0.975$) of the TGA traces following rigorous modeling that involved hyperparameter optimization and testing of the trained model on 20% unseen data. Through a global sensitivity analysis, the degradation temperature for DPB’s thermal devolatilization was identified as the key parameter controlling the pyrolysis process. The techno-economic assessments of the pyrolysis operation indicate that it is a viable, financially rewarding, and environmentally friendly process, offering valuable insights for policymakers, environmental engineers, and energy professionals toward promoting sustainable waste management and a circular economy.

## 1. Introduction

The Kingdom of Saudi Arabia (KSA), along with its neighboring Middle Eastern countries, is actively addressing waste management and landfill challenges by adopting the United Nations’ approaches to the circular economy (SDG 7), specifically through waste-to-wealth initiatives. These initiatives focus on transforming landfill waste into valuable resources and energy (affordable and clean energy). Notable examples of these countries’ commitments to circular economy principles include KSA’s National Waste Management Strategy, Qatar’s plastic recycling efforts, and the United Arab Emirates’ (UAE) investments in waste-to-energy projects [1]. Collectively, these initiatives aim to enhance cost-efficiency in waste-to-energy processes, promote resource recovery, and reduce reliance on fossil-derived energy sources, thereby mitigating the global health crises exacerbated by such dependencies.

Previous research has shown that agricultural byproducts can be processed into fuel derived from biomass, including biogas and syngas [2]. Thermokinetic analysis, which uses data from thermogravimetric analysis (TGA), is frequently used for quantifying the bioenergy potential of these materials [3]. This involves measuring the weight loss of biomass during thermal devolatilization and relating it to degradation temperatures and heating rates. This analysis helps identify possible reaction pathways and their associated rates and energy requirements. For example, one study used dried and pulverized banana peel as a viable material for producing biofuel by way of pyrolysis [3]. The research [3] used Friedman, KAS, and FWO model-free methods to compute the apparent activation energy. The estimated activation energy was reported as 109 ± 9 kJ·mol^−1^, representing the minimum energy needed to thermally degrade the lignocellulosic components of the biomass material into bio-oil, bio-gas, and biochar products. The process involved three main devolatilization steps, each corresponding to the release of key volatile components. Another study by Albin Zaid and Otaru [4] analyzed TGA data from recycled Saudi coffee waste. Their experiments were performed at heating rates of 20, 40, and 60 °C·min^−1^, spanning a temperature range of 25 to 600 °C. The goal was to determine the details of how a reaction could occur, including its thermal and kinetic properties. They reported that the chemical change in thermal degradation of the material occurs via second-order (F2) reaction [4]. By employing the model-free FWO, KAS, and Starink methods, the activation energies were estimated as 92.3 kJ·mol^−1^, 87.3 kJ·mol^−1^, and 87.7 kJ·mol^−1^, respectively.

Kinetic data for the pyrolysis of date palm waste, consisting of leaves, stems, and seeds, were evaluated using TGA system [5]. The thermogravimetric data revealed a two-step degradation process under heating conditions of 10 to 30 °C·min^−1^, consistent with the degradation of the main parts of biomass: hemicellulose, cellulose, and lignin. This stage was succeeded by the gradual oxidation of the remaining char. The estimated energy thresholds for thermal breakdown of the different components of date palm waste were found to be in the range of 92 to 120 kJ·mol^−1^, using the FWO method, and between 86 and 117 kJ·mol^−1^, employing the KAS method. In another experimental TGA analysis [6], the thermal devolatilization of date palm frond yielded estimated activation energies of 84.09 to 145.28 kJ·mol^−1^, determined with the Starink model for varied heating rates: 10, 20, and 40 °C·min^−1^. Falamarz Tahir et al. [7] studied the suitability of date palm trunk and date pits for char production was conducted through TGA analysis from room temperature to 600 °C. The estimated volatile matter in the study ranged between 75.2% and 82.1%, which was attributed to the material’s constituent cellulose, hemicellulose, and lignin. The biomass heating value was also reported to be 20.3 MJ/kg, indicating a relatively high energy density compared to other biomass materials.

Several studies have investigated the bioenergy potential of agricultural wastes and thermogravimetric analysis (TGA). However, few have applied machine learning algorithms to analyze thermal data obtained for the co-pyrolysis of these materials. Typically, Xiao & Zhu [8] utilized deep neural networks (DNN) to model pyrolysis kinetics for 32 different types of biomass materials. Their study [8] revealed that the optimal number of hidden neurons was between 7 and 11, with the highest relative deviations for inputs from the degradation of the main biomass constituents: cellulose, hemicellulose, and lignin. Liu et al. [9] used deep learning models based on ANN, support vector machine (SVM), and random forest (RF) algorithms to model the activation energy values obtained for the pyrolysis of lignocellulosic biomasses. Using 1523 sets of activation energy data from the literature, the RF model was determined to be more precise than the ANN and SVM models. Similarly, Wang et al. [10] used both extreme gradient boosting regression and ANN to predict biomass characteristics and pyrolysis conditions, using 275 data points from various lignocellulosic biomass sources like fruit husk, bamboo, and straw. Their study [10] found that the extreme gradient boosting regression models offered the best predictions, with estimated R-squared ranging between 0.83 and 0.94.

Date palm waste is abundant in Saudi Arabia and the wider Middle East and is a significant source for sustainable bioenergy production via thermochemical or biochemical methods. Hence, understanding the bioenergy potential of date palm branches through experimentation or prediction is vital for implementing climate change initiatives like the Middle East Regional Climate Initiative and the KSA Circular Carbon Economy Model [11], which aims to promote bioenergy, reduce greenhouse gas emissions, and sustainably use agricultural waste. This study investigates the thermal devolatilization of date palm waste, which includes date leaflets and rachis stalks. The research uses a multi-pronged approach combining experimentation, thermokinetic analysis, machine learning (specifically, the k-nearest neighbors’ algorithm) to analyze and optimize experimental conditions along with techno-economic assessments of the process. Few studies have combined machine learning (specifically k-nearest neighbors, or KNN) and thermokinetic to investigate the thermal properties and bioenergy potential of date palm waste; this approach for predicting TGA traces is a novel contribution to the existing literature on machine learning application in thermal analysis.

The unique combination of thermogravimetric measurements, reaction modeling, and machine learning using the K-nearest neighbors (KNN) algorithm in this study is anticipated to provide a comprehensive approach for analyzing the bioenergy potential of date palm branches (DPB). Thermogravimetric analysis (TGA) and derivative thermogravimetry (DTG) measurements are critical in determining the various components of biomass through their specific decomposition temperatures, which influence fuel quality, conversion efficiency, reaction pathways, and the design of efficient thermochemical conversion processes such as gasification and pyrolysis. Furthermore, the conversion of biomass to bioenergy involves complex interactions among its components, often resulting in non-linear data patterns that are challenging to manage. The selection of the KNN machine learning algorithm in this study is based on its simplicity and interpretability, non-parametric and flexible nature, data adaptability, reduced requirement for hyperparameters, and high potential accuracy with sufficient data [12]. Consequently, this study is organized into the following segments:

1. Experimental characterization of the DPB biomass: proximate and ultimate analysis, FTIR and SEM/EDS measurements, and TGA/DTG measurements.
2. Reaction modeling and thermokinetic analysis of the TGA/DTG traces obtained from the thermal devolatilization of the materials.
3. Application of the KNN machine learning algorithm for the prediction and optimal analysis of the thermogravimetric data.
4. Techno-economic assessments (TEA) of the process.

## 2. Materials and Methods

The date palm branch (DPB) utilized for this study was sourced from Al Ahsa located in the Eastern Region of KSA, Saudi Arabia. The DPB biomass waste was collected during the summer months in the KSA, Al Ahsa, Saudi Arabia, during which ambient temperatures ranged from 35 to 50 °C. The dried DPB biomass sample, comprising leaflet and rachis stalk, was cut into pieces and subsequently oven-dried at 50 °C for a period of twelve hours. Subsequently, the dried DPB biomass was pulverized into powder particles with a size distribution of 150–200 µm. This size bracket is critical for assessing the material’s physicochemical properties and functional groups [13], as well as for minimizing any effects imposed by thermal contact surfaces resulting from broader particle size variations or multi-modal arrangements [14]. The estimated density of the pulverized DPB sample following tapping of the material (tapped density) is 0.47 g/cm^3^, while the apparent density is 0.39 g/cm^3^. This relatively low apparent density can be credited to the transversely isotropic filamentous structure of the material, which results in significant air gaps within the fibrous structure [15]. To support the research, powdered DPB biomass was put through a series of tests, including compositional (proximate and ultimate), spectroscopic (FTIR), microscopic (SEM/EDX), and thermogravimetric analysis (TGA).

### 2.1. Compositional Analysis

The proximate analysis of the pulverized DBP biomass sample composition was conducted using a Mettler Toledo TGA system, following a procedure like those described in previous studies [16,17]. This analysis examined the impact of temperature variations on weight loss in an oxygen-free atmosphere to ascertain the proximate composition (moisture, fixed carbon, volatile matter, and ash contents) of the sample. A sample weighing 9 mg was accurately measured and placed in the crucible of the TGA system. The crucible was then positioned in the heating chamber of the TGA analyzer and weighed. The DPB biomass sample was heated at a rate of 10 °C·min^−1^ starting from room temperature and continuing to 900 °C, within an inert atmosphere. Upon initiation of the process, weight changes at a constant temperature of 105 °C for a duration of 1 h and 30 min were utilized to determine the moisture content of the sample. The temperature subsequently increased to 600 °C for 8 min to measure volatile matter. The total ash yield of the pulverized DBP biomass sample was quantified at temperature of 900 °C for 1.5 h. Fixed carbon was obtained by calculating the residual percentage after accounting for moisture, ash, and volatile matter. It is crucial to highlight that the TGA procedure described here is different from the procedure required for kinetic analysis. The key difference lies in their respective experimental conditions and estimated data. Typically, proximate analysis uses a stepwise temperature program to mimic conventional laboratory techniques that use a furnace or pyrolyzer for such a process, while kinetic TGA studies require controlled heating rates to calculate devolatilization thermokinetic parameters (to be discussed later).

A CHNS-O elemental analysis of the DPB biomass sample was performed using a dual carrier VELP Scientifica EMA 502 elemental microanalyzer (Usmate, Italy), following the procedure outlined in [18]. Through a high-temperature combustion procedure, the elemental makeup of the DPB biomass is quantified, providing the percentage breakdown of C, H, N, S, and O. A 2.2 mg sample of the pulverized DPB biomass was securely encased in an aluminum crucible, which was subsequently placed into the analyzer. Preheating of the sample was accomplished by introducing helium gas flowing at 120 mL·min^−1^ and 900 mbar of pressure, with gas chromatography and thermal conductivity detector (TCD) temperatures maintained at 57 ± 3 °C. Oxygen gas was then purged into the combustion chamber of the elemental analyzer to facilitate the conversion of the elements into gaseous compounds. Specifically, carbon is converted to CO_2_, hydrogen to H_2_O, nitrogen to N_2_, and sulfur to SO_2_. The integrated gas chromatography detector within the CHNS analyzer measures the volumetric percentages of each of the four gases (C, H, N, S), while the percentage of oxygen content is calculated through a process of subtracting the sum of the volumetric percentages of the CHNS gases from 100%. The CHNS data were recorded using the VELP EMA Soft^TM^ data acquisition software.

### 2.2. FTIR and SEM/EDS Measurements

Fourier Transform Infrared (FTIR) spectroscopy of the pulverized DPB materials was conducted to identify the organic compound present within the sample. The analysis was conducted using a Cary 630 FTIR spectrometer equipped with Agilent MicroLab Expert software (version 5.8), following a procedure similar to that described in the cited reference [19]. Prior to the FTIR experiment, acetone was employed to clean the crystal surface of the FTIR system, owing to its strong solvency, bipolar and hydrophilic characteristics, and rapid evaporation properties [20]. This cleaning process facilitated the effective and timely removal of contaminants from the crystal surface that could interfere with infrared light absorption, potentially leading to inaccurate spectral results. A background measurement of the crystal (without a sample) was obtained to provide a reference for comparison when a sample was placed on the crystal surface. The sample was subsequently positioned on the crystal position of the FTIR spectrometer and securely held in place using the press button, after which infrared transmissivity is absorbed by the sample and recorded in the software. Following the completion of data collection, the spectral interpretation was analyzed with the software and exported as a Microsoft Excel file.

The surface topography, particle dimensions, and chemical/elemental makeup of the pulverized raw DPB biomass material and the thermally decomposed biochar were analyzed using a benchtop scanning electron microscope (Joel JCM-7000 NeoScope SEM). The pulverized DPB biomass specimen was secured on the SEM stub and made conductive by sputter-depositing a gold–palladium (Au-Pd) with a Quorum QR150R. The application of the gold–palladium coating is critical for preventing charge accumulation, which can distort imaging and affect analytical results, as well as for enhancing the secondary electron signal, resulting in improved image resolution and contrast [21]. The electron beam of the SEM system scans the surfaces of the coated sample, producing signals from backscattered electrons and the excitation of atoms. This process forms the basis for the SEM images, which provide detailed information regarding the sample’s surface morphological features, shape, dimensions, and captures X-ray energies that are converted into a spectrum identifying the present elements and their relative abundances [22]. This elemental analysis of the sample is referred to as energy-dispersive X-ray spectroscopy (XEDS). The SEM/XEDS analysis results obtained for the DPB were systematically extracted and analyzed.

### 2.3. TGA/DTG Measurements

Thermogravimetric analysis (TGA) of the pulverized DPB biomass sample was undertaken utilizing a thermal analysis system (Mettler Toledo TGA/SDTA 851e thermogravimetric analyzer), with temperature ranges extending from room to 1000 °C and heating rates of 7.5, 15, 30, and 60 °C·min^−1^. A sample weight of the pulverized material was placed in the crucible of the TGA, ensuring that the sample entirely covered the bottom of the crucible to mitigate heat gradients and optimize the elution of volatiles [23,24,25]. The crucible containing the sample was positioned within the heating chamber of the TGA system, followed by the introduction of nitrogen gas flow (20 mL·min^−1^) into the furnace chamber of the thermal analyzer, thereby ensuring an oxygen-free environment. The TGA data acquisition software (STARe Software Version 15.00, Build 8668) was initially configured to obtain TGA data at a 7.5 °C·min^−1^ of heating rate, with varying temperature between 25 °C and 1000 °C. Upon initiation of the experiment, sample weight loss and degradation temperature were recorded, with the duration of the experiment influenced by the chosen heating rate. After reaching 1000 °C, the sample and thermal analyzer were air-cooled to room temperature before subsequent tests to ensure instrument stability and prevent premature reactions from residual heat, which guarantees reliable, reproducible, and accurate TGA data. Further TGA analyses were conducted twice for all heating rates and varying temperature ranges, resulting in differences in TGA traces within a range of ±3 percent in weight loss when compared. Derivative thermogravimetric (DTG) rate profile for the thermolysis operation of the DPB sample were calculated using weight loss and temperature data (i.e., the TGA traces) obtained from experimental measurements.

### 2.4. Solid-State Reaction Mechanisms and Thermokinetic Methods

The methodologies for estimating the kinetic triplet, which encompasses three fundamental parameters—reaction mechanism (or kinetic model characterizing the chemical reaction), activation energy ($E_{A}$) and frequency factor or pre-exponential factor (A)—as well as thermodynamic variables including activation enthalpy (∆H), Gibbs free energy of the activated complex (∆G), entropy of activation (∆S), and equilibrium constant (k), are delineated in this section. In this study, the kinetic triplet plays a critical role in understanding the thermal devolatilization characteristics of DPB sample and in optimizing reaction conditions, as evidenced by the decomposition of various material compositions from room temperature to ash [26]. The Criado master plots were employed in this section of the study to identify the solid-state reaction mechanisms (theoretical model functions) that fully characterize the experimental curve. The master plot model in Equation (S1) (supplementary information) shows the reduced experimental $\left\{{\left(\frac{T_{x}}{T_{0.5}}\right)}^{2}\frac{{\left(dx/dt\right)}_{x}}{{\left(dx/dt\right)}_{0.5}}\right\}$ and theoretical $\left\{\frac{f\left(x_{i}\right).g\left(x_{i}\right)}{f\left(0.5\right).g\left(0.5\right)}\right\}$ mathematical relations that are plotted against the conversions from 0 to 1 to determine the possible mechanism(s) of thermochemical conversion. Table S1 (supplementary information) presents 20 selected solid-state reaction mechanisms categorized into four distinct groups: diffusion, geometrical contraction, reaction order, and sigmoidal rate.

Biomasses are primarily characterized as complex mixtures of substances, and their thermal devolatilization often results in multiple overlapping differential thermogravimetry (DTG) traces and peaks [27], which reflects the breakdown of the biomass compositions: cellulose, hemicellulose, and lignin components (to be discussed later). Due to the complexity resulting from the thermal devolatilization of the biomass materials, the direct application of the Criado master equation across the entire degradation temperature range of 25 to 1000 °C may yield inaccurate reduced theoretical traces [28], which may not completely overlap with experimental DTG traces. Consequently, deconvolution kinetic analysis was applied to the DTG data using Bi-Gaussian functions (coded in MATLAB^TM^ R2024a) to isolate unique decomposition ranges relating to the pseudo-components of lignocellulosic DPB biomass [29,30]. This approach provides perspective on the estimation of potential decomposition pathways and corresponding kinetic parameters. The selection of the Gaussian mathematical function for the deconvolution of the DTG traces in this study is based on its adaptability in mean and width to reflect a variety of real-time data [31], and it has been deemed to offer a more precise representation of kinetic variables [30].

Kinetic data, including activation energy and frequency factor obtained from the formation of activated complexes during pyrolysis of pulverized DPB sample, were assessed utilizing the Flynn–Wall–Ozawa (FWO) [32,33], Kissinger–Akahira–Sunose (KAS) [34,35], Starink (STK) [36], and Friedman (FR) [37,38] model-free kinetic methodologies. Although model-fitting approaches like the Borchardt–Daniels [6] and Coats–Redfern [4] models have been documented in the existing literature, the selection of model-free methods in this investigation is informed by the ICTAC reports in [28], which underscore the superior reliability of model-free methodologies in the acquisition of kinetic data compared to model-fitting techniques. The preference for model-free methods in [28] was substantiated using multiple heating rates (minimum of three) for the estimation of kinetic parameters, thereby ensuring reliability [38] and recommending the application of model-free kinetic methods for the precise estimation of activation energy and thermodynamic data. Equations (S2)–(S5) (supplementary information) define the mathematical expressions for the FWO, KAS, STK, and FR model-free kinetic methods. Additionally, the thermodynamic parameters, including the activation enthalpy (∆H) [39], entropy of activation (∆S) [40], Gibbs free energy of the activated complex (∆G) [39,40], and the equilibrium constant (*k*) [39], associated with the thermal devolatilization of the DPB biomass material were estimated using the mathematical relations specified in Equations (S6), (S7), (S8) and (S9), respectively (Supplementary Information).

### 2.5. Application of K-Nearest Neighbors to TGA Data

The k-nearest neighbors (abbreviated as KNN) algorithm is classified as a supervised learning technique frequently employed for modeling both numerical (regression) and categorical (classification) problems. KNN operates by identifying the “k” data points that are closest to an unknown data point and subsequently utilizes the information from these “k” nearest neighbors to predict the value (in the case of regression) or the class (in the case of classification) of the new data point [41]. The selection of “k” serves as a hyperparameter in the learning process during training and can significantly influence the model’s performance; specifically, underestimating “k” often results in overfitting, while overestimating “k” may smooth out decision boundaries, potentially leading to underfitting by losing fine-grained details [42]. Consequently, the determination of the k-value is critical to the accuracy of the model, thereby enhancing performance for both training and unseen data (testing data). The machine learning algorithm was chosen for analyzing TGA traces of DPB biomass pyrolysis because it is simple, manages non-linear relationships without complex assumptions [43], and is valuable for accurate outcome estimation and process optimization.

The implementation of the k-nearest neighbors (KNN) algorithm herein commenced with preparing data for analysis prior to its importation into MATLAB™ for machine learning analysis. Approximately 396 experimental data points were culled from TGA data, corresponding to four heating rates and temperatures ranging from 25 to 1000 °C. The extraction process involved selecting the initial data points at 25 °C and subsequently selecting data points from 30 to 1000 °C with a temperature increment of 10 °C. The dataset was meticulously examined to ensure the absence of outliers and uniformity in dimensions. The extracted features, which included degradation temperature, heating rates, and time, along with the label (TGA sample weight), were imported into MATLAB, followed by a division of the data into training/validation and testing. An 80/20 data-splitting strategy was employed, reserving 20% of the dataset for unbiased performance evaluation after the model was trained on the remaining 80% (training/validation). Consequently, approximately 317 data points were utilized for training/validation, leaving 79 data points for testing. This data-splitting strategy is essential for ascertaining the model learns general patterns instead of rote memorization, from the entire dataset, thereby mitigating the risk of overfitting [44]. Normalization of the input features was conducted through Z-score standardization, transforming them to have a mean of 0 and a variance of 1 [45]. Since KNN is a distance-based algorithm [45], minimizing the influence of larger numerical values over smaller ones is critical for achieving accurate predictions. The K-nearest neighbors (KNN) model was trained using MATLAB’s ***fitcknn*** function. An initial k-value of 23 was selected, determined by the next odd integer following the square root of the total number of data points. This selection was subsequently followed by hyperparameter optimization and the extraction of modeled data for analysis.

## 3. Discussion of Results

This section provides a detailed presentation and analysis of research data gathered through experimentation and predictive modeling. The analysis is structured into several key categories:

- **Proximate and ultimate analysis:** Analysis of the fundamental breakdown of material’s composition.
- **Analysis of SEM/XEDS and FTIR data:** Detailed analysis of material characterization using advanced microscopic and spectroscopic techniques.
- **TGA/DTG data:** Analysis of material decomposition under controlled heating.
- **Analysis of thermokinetic data:** Analysis of the reaction, kinetics and thermodynamics of the process.
- **Application of K-Nearest Neighbors to TGA Data:** The use of machine learning KNN to interpret thermal analysis results.

### 3.1. Proximate and Ultimate Analysis Data

Table 1 presents the material’s elemental and compositional breakdown of the pulverized DPB biomass, respectively. The moisture, volatile matter, fixed carbon, and ash contents of the sample were estimated to be 4.47%, 18.14%, 72.01%, and 5.38%, respectively. The sample’s moisture content is advantageously low (within the 5–10% range), requiring less energy for drying and benefiting heat-driven processes like incineration, carbonization, and pyrolysis [46,47]. The estimated values of volatile matter and fixed carbon contents for the DPB align with those typically expected of lignocellulosic materials, indicating ease of ignition and high combustion and energy output potential for such materials. Raw lignocellulosic materials typically exhibit 65–85% volatile matter [48] and 10–30% fixed carbon content [49], with variations based on moisture and ash content. The pulverized biomass sample, specifically, had a 5.38% ash content, which is within the optimal range (5–9%) for herbaceous biomass [50], a characteristic beneficial for maximizing combustion efficiency, energy yield, and minimizing fouling.

Table 1 also demonstrates that the pulverized date palm branch (DPB) material is characterized by high carbon and oxygen contents, primarily attributable to its organic compositional nature. This characteristic may enhance its calorific value and char formation, rendering it a valuable energy source [51]. The low concentrations of hydrogen and nitrogen, along with the absence of sulfur in the DPB biomass materials, presents environmental advantages [52] when utilized as a fuel source, lowering carbon footprint and air pollution in comparison to fossil fuels. Nasir et al. [53] estimated the carbon, hydrogen, nitrogen, and oxygen (CHNO) composition of palm frond midrib (rachis stalk) obtained from Riyadh, KSA, as 46.50%, 5.95%, 0.27%, and 48.13%, respectively. Additionally, the reported values [53] for volatile matter, ash, and fixed carbon were 82.28%, 3.56%, and 14.15%, respectively. Falamarz Tahir et al. [7] assessed the CHNO compositions of date palm leaves sourced from Alkhargoulia (approximately 20 km from the city of Baghdad, Iraq) as 42.02%, 6.24%, 0.54%, and 41.20%. The corresponding values for volatile matter, ash, and fixed carbon were recorded at 75.96%, 8.18%, and 11.86%, respectively. The study’s compositional analysis of DPB biomass aligns with estimated values for general date palm waste constituents. It is important to note, however, that slight variations in composition may occur based on the source location, climate, and specific cultivar examined.

### 3.2. Analysis of SEM/XEDS and FTIR Data

**Figure 1:**
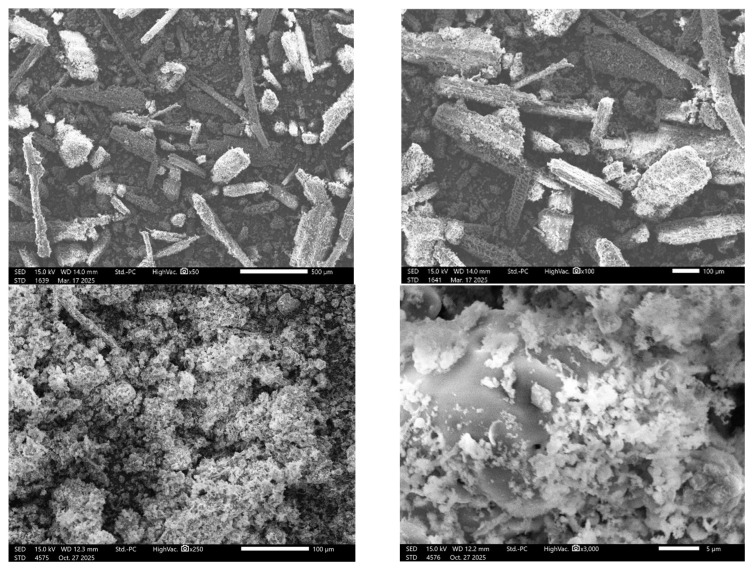
SEM micrographs showing the powdered raw DPB biomass (**top**) and the biochar produced from it following thermal processing (**bottom**).

Figure 1(top) displays SEM surface topology of the pulverized DPB biomass sample, illustrating its porous and heterogeneous surface morphology. The SEM images reveal noodle-like and rod-like structures, indicating the presence of fibers (rachis stalk) as well as sub-rounded and sub-angular particles, which suggest the presence of pulverized leaflets within the material. The presence of the stringy morphological characteristic in the pulverized date palm bract (DPB) material can be attributed to the plant’s inherent composition of strong, long lignocellulosic fibers [54], which support its structural rigidity and stability. Figure 1(top) shows that the “noodle-like” shaped material in the pulverized DPB biomass promotes more porous aggregated structures, creating space for air. This, in turn, could act as a thermal insulator and slow the decomposition process when compared to the more readily heated sub-rounded and sub-angular particles. The estimated particle sizes for the fiber cross-section and particle diameter range between 150 and 200 µm, highlighting porous features that reflect the inherent structure of lignocellulosic materials [55]. Figure 1(bottom) presents the SEM morphology of the biochar produced from thermally induced DPB biomass material at 600 °C. The figure illustrates a highly porous, irregular, and heterogeneous surface structure, where the original morphology of the raw DPB biomass was significantly altered by the pyrolysis temperature. This highly porous nature and open pore networks of the biochar indicate the material could be valuable for applications in adsorption, processing of bio-composite materials, and soil amendment.

Table 2 presents the energy-dispersive X-ray spectroscopy (XEDS) analysis of the pulverized raw DPB biomass material, revealing its elemental composition, which typically consists of high amounts of carbon (C) and oxygen (O), along with varying percentages of mineral elements. The considerable presence of C and O can be attributed to the lignocellulosic nature of the DPB fibrous material [56]. The XEDS data for the biochar composition presented in Table 2 indicates that the presence of silica (Si, approximately 6.81%), chlorine (Cl, approximately 2.53%), potassium (K, approximately 2.79%), and calcium (Ca, approximately 2.53%) in the raw DBP interact at the elevated temperatures associated with its thermal devolatilization. Specifically, potassium (K) and chlorine (Cl) volatilize as potassium chloride (KCl) [57]. Meanwhile, silica sand may react with potassium carbonate (K_2_CO_3_) to form potassium silicates such as kalsilite (KAlSiO_4_), leucite (KAlSi_3_O_8_), and potassium water glass (K_2_O·SiO_2_) [58]. The observed reduction of carbon in the thermally degraded material indicates that volatile matter is lost during the process, while calcium, a non-volatile mineral component, becomes a larger proportion of the remaining solid materials, as shown in Table 2. This interaction contributes to ash formation and fouling [58] during the pyrolysis operation. Trace amounts of aluminum (Al~0.31%), magnesium (Mg~0.62%), sulfur (S~0.39%), iron (Fe~0.89%), and niobium (Nb~0.67%) are present, which are unlikely to significantly influence the reaction pathways or act as natural catalysts in the thermal degradation of the DPB biomass material. Additionally, Table 2 indicates a significant deposit of gold (8.55%) in the pulverized DPB biomass, which can be attributed to the gold-palladium alloy coating applied to the sample during SEM analysis; this coating does not influence the thermal devolatilization process, as the raw DPB used for thermochemical conversion was uncoated.

**Figure 2:**
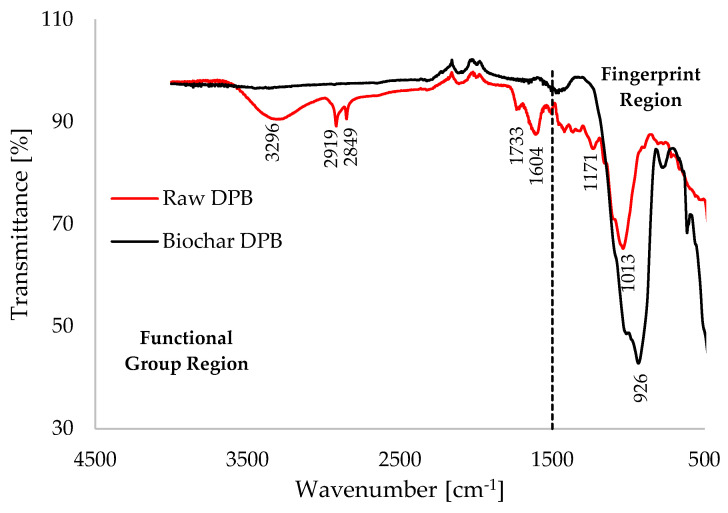
FTIR spectrum of the DPB biomass, illustrating percentage transmittance [%] against wavenumber [cm^−1^].

Figure 2 presents the FTIR spectrum of the pulverized raw DPB biomass, revealing absorption peak characteristics of the various functional groups that are present in the sample. Consistent with the FTIR functional group database [59], the peak at 3296 cm^−1^ belongs to the absorption band of 3200–3550 cm^−1^, which is classified as hydroxyl (O–H) stretching vibrations within the cellulose and hemicellulose components of the DPB biomass material. The sharp peaks at 2919 cm^−1^ and 2849 cm^−1^ correspond to the asymmetric and symmetric stretching vibrations of aliphatic C–H bonds, respectively [60]. The peak at 1733 cm^−1^ is indicative of unconjugated carbonyls, commonly found in polysaccharides such as xylans and other hemicelluloses [61] present in the DPB biomass. Meanwhile, the sharp peak at 1604 cm^−1^ is indicative of C=C bonds within the aromatic rings, which are key components of lignin [62]. The sharp peaks at 1171 cm^−1^ and 1013 cm^−1^ may fall within the fingerprint region—often considered inadequate for basic molecular identification due to its complex “forest of peaks” [63]—but could indicate the presence of C–O–C vibrations of polysaccharides such as hemicellulose and cellulose, as well as C–OH (alcohol) stretching vibrations [59], also found in polysaccharides and herbaceous plants.

### 3.3. Analysis of TGA/DTG Data

**Figure 3:**
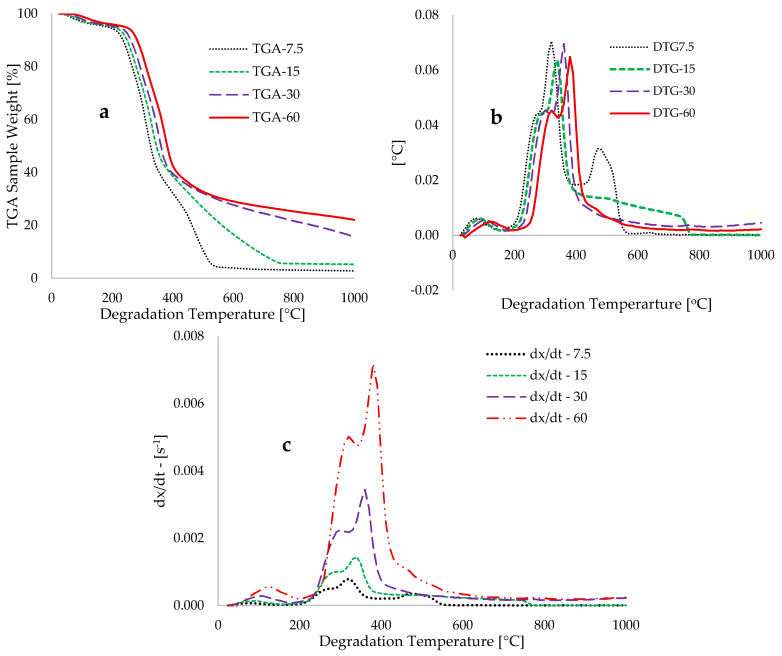
Graphs depicting the plots of (**a**) TGA sample weight [%], (**b**) DTG (−dW/dT, [mg/°C]), and (**c**) dx_i_/dt [s^−1^] against degradation temperature (T, [°C]) at different heating rates.

Figure 3a depicts the TGA traces for the pyrolysis of DBP material at 7.5, 15, 30, and 60 °C·min^−1^ heating rates and temperatures between 25 and 1000 °C. Figure 3b presents the derivative thermogravimetry (DTG) derived from the TGA data, while Figure 3c illustrates the conversion per unit time of thermal decomposition of the sample relative to degradation temperature. Thermogravimetric (TG) and DTG peaks shift to higher temperatures at faster heating rates due to thermal lag and reduced devolatilization residence time [63]. The DTG traces in Figure 3b indicate that the initial degradation peaks occurred at temperatures between 40 and 220 °C, with an increase in temperature corresponding to higher heating rates. This initial degradation accounts for approximately 5 wt% of the sample and is attributable to the loss of moisture content from the date palm branch biomass, as detailed in the compositional data presented in Table 1. The subsequent degradation bands occurred within 200 to 530 °C, representing the primary degradation stage associated with the loss of hemicellulose and cellulose from the fibrous material. Referring to the DTG data obtained at a constant heating rate of 30 °C·min^−1^, the peaks for hemicellulose and cellulose decomposition were observed at 310 °C and 360 °C, respectively, accounting for losses of 14.18 wt% and 45.18 wt%, respectively. Nasser et al. [53] reported values of 16.13 wt% and 47.14 wt% as the percentage compositions of hemicellulose and cellulose in date palm leaflets, respectively, with composition ranges recorded as 17 to 34 wt% for hemicellulose and 34 to 49 wt% for cellulose across various parts of date palm waste [64]. Additionally, the study referenced in [64] reported degradation temperature ranges for hemicellulose and cellulose within fibrous materials as 200 to 350 °C and 280 to 370 °C, respectively.

Figure 3a illustrates that the degradation of lignin decelerates under high heating conditions, specifically at rates exceeding 30 °C·min^−1^ and temperatures above 470 °C. In contrast, degradation at 7.5 and 15 °C·min^−1^ persists until temperatures reach 540 °C and 740 °C, respectively, ultimately resulting in ash formation. Figure 3c demonstrates that elevated heating rates primarily facilitate the thermal devolatilization of hemicellulose and cellulose components within DPB biomass. The complex and heterogeneous structures, as well as the stronger bonds associated with lignin, necessitate its slower degradation under these elevated temperature and heating rate conditions [65]. Although lignin degrades more slowly than hemicellulose and cellulose, it exhibits more rapid thermal degradation at lower heating rates. This happens because slow heating rates often lead to a complete and more efficient breakdown of the hemicellulose and cellulose components of the DPB fibrous material. Consequently, the lower temperatures promote the breakdown (de-carbonylation and de-methylation) of ether bonds in lignin, leading to the formation of smaller aromatic molecules (oligomers and phenols) [66], resulting in more depolymerized lignin that degrades more rapidly. Figure 3c corroborates the findings presented in [67], supporting the characterization of lignin as a rate-limiting factor in the pyrolysis of lignocellulosic biomasses, which is inversely correlated with the degradation rates of cellulose and hemicellulose.

### 3.4. Analysis of Thermokinetic Data

**Figure 4:**
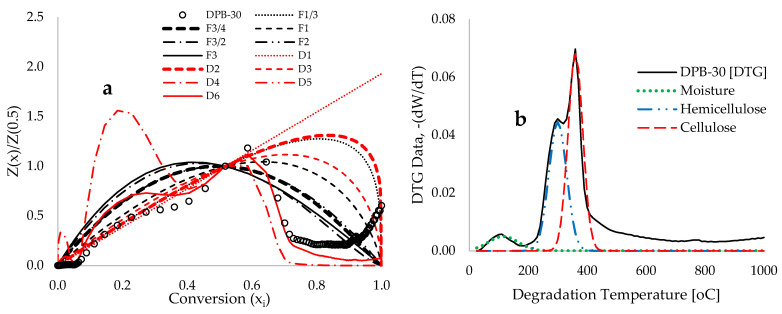
Graphs depicting (**a**) Criado master plots that present reduced experimental and theoretical data at a constant heating rate of 30 °C·min^−1^, estimated between 25 and 1000 °C, and (**b**) both “real” and “deconvoluted” DTG traces illustrating distinct peaks associated with dehydration, hemicellulose decomposition, cellulose decomposition, and the gradual degradation of lignin.

Figure 4a illustrates a prior representation of the Criado master plot, showcasing reduced experimental and theoretical plots estimated through the reaction-order and diffusion models, plotted against conversion prior to the application of the deconvolution technique. Notably, this figure shows significant deviations between the experimental and analytical-derived traces, which may be attributed to variations in the compositions of DPB biomass. Consequently, the thermal decomposition of this material is complex, leading to multiple simultaneous reactions, multi-step degradation peaks, diffusion processes, and char formation [68]. In contrast, the reduced theoretical models were developed to capture behavior characterized by a single-step reaction mechanism. Figure 4b presents the deconvoluted DTG traces for moisture, hemicellulose, and cellulose during pyrolysis operations conducted at 30 °C·min^−1^, with peaks and widths overlapping with experimental data, while slow lignin decomposition was observed without a distinct peak. The deconvoluted peaks, derived using least-squares algorithms and Gaussian functions based on DTG parameters, accurately represent the complex decomposition of DPB biomass. This approach segregates the DTG data into distinct “pseudo-components” [69], which uncover the possible reaction pathways and thermokinetic data pertinent to the pyrolytic operation.

**Figure 5:**
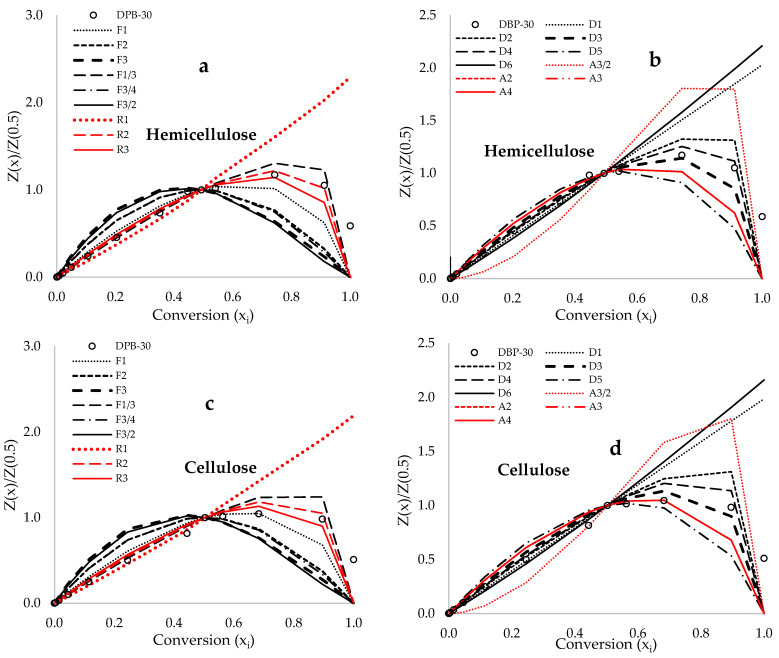
Criado master plots denoting both the reduced theoretical and deconvoluted experimental Z(xi)/Z(0.5) in conjunction with the theoretical data obtained at a constant heating rate of 30 °C·min^−1^ against conversion (x_i_).

Figure 5a–d display the experimental and theoretical curves for the hemicellulose and cellulose components within date palm biomass across conversion rates of 5% to 70%. These plots demonstrate that the R3 contracting cylinder of the geometrical contraction model (GCC) offers the most accurate fit for the experimental curves relating to the decomposition of hemicellulose and cellulose, followed closely by the Jander [D3] and Ginstling–Brounshtein [D4] diffusion models. The assessment of this thermal decomposition mechanism of DPB is critical for the precise determination of the thermokinetic parameters of the process, thereby facilitating the optimization of operational parameters like product yields, temperature and heating rate [70]. Furthermore, this understanding aids in the design of chemical reactors for pyrolysis and gasification operations. In contrast to the existing literature, Hai et al. [71] reported that the Zhuravlev [D5] equation of the diffusion model most accurately characterized the pyrolysis of date palm waste, particularly date pits. In a related investigation by Raza et al. [72], both the Ginstling–Brounshtein and the Ginstling diffusion equations of solid-state reaction mechanisms were identified as the more accurate models for the thermal devolatilization of date palm surface fibers. Moreover, information from [71,72] indicates that the TGA traces obtained for other forms of date palm waste exhibit a comparable trajectory, with minor deviations in the estimated data. Factors like particle size, heating rates, and the specific part of the date palm analyzed (this study considers the combined rachis stalk and date leaflet) may significantly influence the pyrolysis reaction [72].

**Figure 6:**
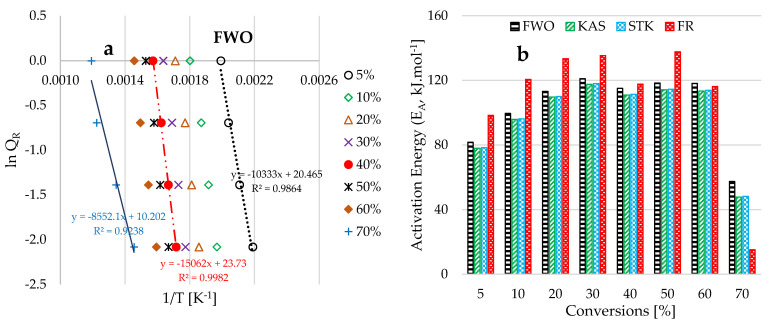
Graphs illustrating (**a**) the estimated data resulting from the application of the FWO kinetic method against the inverse of temperature [K^−1^] for conversions between 5% and 70%, and 30 °C min^−1^ of heating rate; and (**b**) the estimated activation energy data against conversions.

Figure 6a presents plots of *lnQ_R_* against the inverse of temperature for the heat-induced operation conducted at a constant heating rate of 30 °C·min^−1^, and conversions ranging from 5% to 70%. It is crucial to emphasize that the range of conversions for kinetic study was determined by the spacing of the TGA traces and their approach to a plateau at high temperatures. For instance, at elevated temperatures and high heating rates typically associated with conversions exceeding 70%, the close spacing of the TGA traces can pose challenges for interpretation, potentially resulting in inaccurate estimations of temperature and transformation rates. Furthermore, in Figure 3a, at elevated heating rates and temperatures—specifically beyond 600 °C—the TGA traces nearly plateau. Consequently, selecting temperatures and conversions beyond this region may necessitate the utilization of experimental data obtained at lower heating rates, which is insufficient for the accurate determination of thermokinetic parameters [38,73]. A meticulous examination of the plots in Figure 6a reveals that the linear inverse trends exhibit distinct differences across the range of conversions from low to high. For example, the inclined line corresponding to 5% conversion is markedly separated from the other slanting lines, which may be attributed to the significant energy change occurring between the dehydration and propagation stages of the pyrolysis of DPB biomass. The closely spaced slanting lines obtained for conversions between 10% and 60% can be ascribed to the overlapping degradation of hemicellulose and cellulose components of the biomass, whereas the inclined line for lignin degradation at 70% conversion diverges from the other slanting lines. Accordingly, the slopes and intercepts of these slanting lines are incorporated into the kinetic methods described in Equations (S2)–(S4) (supplementary information) to account for the activation energy ($E_{A}$) and pre-exponential factor (A) associated with the thermal devolatilization of DPB biomass.

Figure 6b presents the estimated activation energy data obtained for conversions ranging from 5% to 70% across each of the selected model-free kinetic methods: FWO, KAS, STK, and FR. The data in Figure 6b indicates high estimated activation energies during the propagation stage, primarily due to the devolatilization of hemicellulose and cellulose. This elevated activation energy associated with hemicellulose and cellulose degradation can be credited to the strong linear (cellulose) and branched (hemicellulose) crystalline polymer structures, which possess strong intermolecular and intramolecular hydrogen bonds [74]. These attributes result in a stable and rigid structure that necessitates greater energy input for dissociation and disruption, particularly when compared to the processes of dehydration and lignin degradation. A comparison of the figures shows that the calculated activation energy largely depends on the spacing between the TGA traces at different heating rates.

Figure 6b illustrates that the estimated activation values obtained using the FWO, KAS, and STK models are closely aligned. These three models are classified as model-free integral methods because they are predicated on the principle that the reaction rate is primarily dependent on the degradation temperature at a given conversion level [75]. Furthermore, Figure 6b and the summarized statistical data in Table 3 show that higher activation energy values were estimated using the Friedman (FR) model-free kinetic method. The FR method employs differential mathematical approximations, which introduce inherent levels of error and assumptions into the calculation of activation energy [76], in contrast to the model-free integral methods. Table 3 indicates that the activation energy values calculated using the FR model are consistently higher and have a greater range of uncertainty compared to those derived from the model-free integral methods. This broader range in the FR model results in less precise estimates. The negative estimated values of skewness indicate an asymmetrical distribution, with the median values exceeding the mean values of the activation energy data—this discrepancy is most pronounced in the FR method, which exhibits the most negative skewness. Additionally, the high kurtosis (leptokurtic) value obtained for the FR method signifies a distribution characterized by heavy tails and a sharp peak [77], suggesting an increased likelihood of extreme values or the presence of outliers, thereby rendering the estimated activation energy values obtained through this model less reliable. Consequently, the tabulated statistical data in Table 3 provide strong support for the assertions made in [32,78] that the FR model is more sensitive to outliers (data noise). In the context of more complex reactions and the multistep degradation of DPB biomass, this presents a critical challenge that can introduce errors into the analysis.

Table 4 summarizes the estimated thermokinetic data for the thermal degradation of date palm branch (DPB) biomass materials, utilizing model-free integral methods that exhibit superior coefficients of determination. The mean values of activation energy derived from these kinetic methods range from 98.43 to 103.06 kJ·mol^−1^, whereas the calculated activation energy using the FR kinetic method is 109.30 kJ·mol^−1^. Comparatively, Alsulami et al. [5] reported activation energies for date palm waste obtained from the utilization of the FWO and KAS model-free integral methods, estimating values between 92 and 120 kJ·mol^−1^ and 86 and 117 kJ·mol^−1^, respectively. Similarly, Raza et al. [72] provided activation energy estimates of 92 to 120 kJ·mol^−1^ and 86 to 117 kJ·mol^−1^ using the FWO and KAS isoconversional methods for date palm surface fibers. Consequently, the overall activation energy estimated for the date palm branch in this study falls within the established range reported for various date palm waste materials. This value aligns with the lower limit of the realistic activation energy range (70 to 270 kJ·mol^−1^) typically expected for most biomass pyrolysis operations [79,80], indicating that initiation of the pyrolysis requires minimal energy input. This characteristic results in a faster reaction rate, classifying DPB biomass as a softer biomass material. The theoretical gross calorific value (GCV) calculated using the fixed carbon content presented in Table 1 is 17.67 MJ/kg, which is notably higher than that of several other biomass materials. This elevated GCV, combined with reduced moisture content and lower activation energy, serves as positive indicators that position DPB as a viable pyrolysis feedstock and a promising source of bioenergy.

Table 5 presents the estimated overall values of activation enthalpy and Gibbs free energy of the activated complex, ranging from 93.15 to 104.03 kJ·mol^−1^ and 180.47 to 279.06 kJ·mol^−1^, respectively, for the four selected model-free methods. The estimated activation enthalpies are quite close to the estimated values for the overall activation energy, with an average difference of 5.27 ($R.T_{M}$) kJ·mol^−1^, which accounts for the work required to expand the volume during the formation of the transition state. This minimal difference in activation energy ($E_{A}$) and activation enthalpy (∆H) suggests that the energy barrier required to initiate the chemical reaction during the thermal devolatilization of DPB biomass is primarily dependent on heat changes rather than on changes in molecular disorder [81]. It is also indicated that the entropy of activation (∆S) is close to zero [81], as supported by the estimated values in Table 4, which suggest that the transition state is highly ordered, exhibiting minimal vibrational or rotational degrees of freedom compared to the reactants [39,82]. The calculated Gibbs energy of the transition state (∆G), derived from the four selected model-free kinetic methods, are all positive and fall within the expected range for biomass conversion. This suggests that the attainment of the transition state configuration (activated complex) is non-spontaneous and will necessitate an external energy input to proceed [39]. Furthermore, the relatively low estimated values of the equilibrium constant (k) for the pyrolysis of DPB biomass, as shown in Table 4, imply that at equilibrium, the pyrolysis of the material is reactant-favored or represents a highly endergonic reaction [81]. In summary, the pyrolysis of DPB biomass is an endothermic process that requires a continuous input of energy to initiate and sustain the reaction.

### 3.5. KNN Analysis of TGA Data

**Figure 7:**
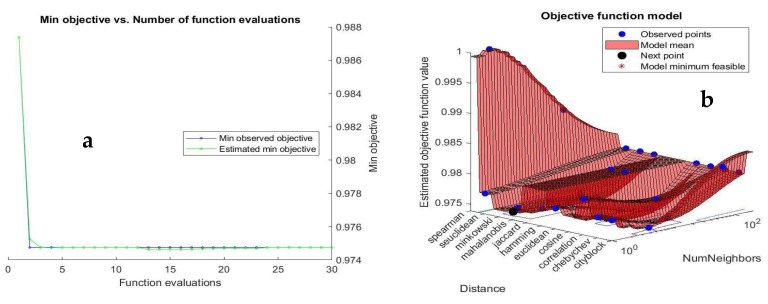

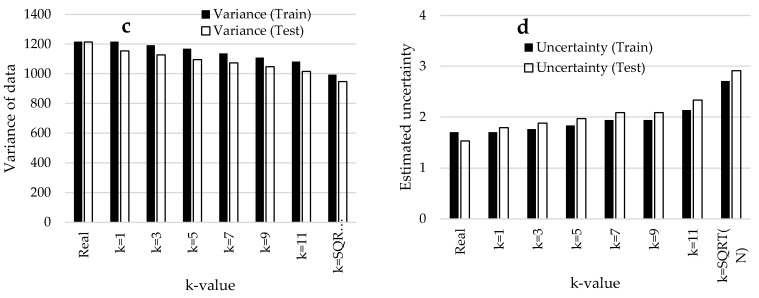
Graphical representations illustrating (**a**) the minimum objective value versus as a function of the number of function evaluations for a k-value of SQRT(N), (**b**) a 3D plot of optimized hyperparameters demonstrating the changes in the objective function resulting from variations in the number of neighbors and different types of distance metrics for a k-value of SQRT(N), and bar charts depicting (**c**) the variance of the data and (**d**) the estimated uncertainty against the k-value for both the training and testing datasets.

Figure 7a illustrates the minimum objective function, or training loss, against the number of function evaluations, termed epochs. The data indicates that an increase in function evaluations correlates with a decrease in the objective function during training, demonstrating convergence towards a minimum. The optimization of hyperparameters was performed to identify the appropriate k-value (number of neighbors) and distance metric, facilitating the attainment of a prompt and satisfactory local minimum that is close to the global maximum [83,84]. Figure 7b depicts the estimated objective function value in relation to the average value of the k closest data points (k-value) and various distance metrics, including Euclidean, Minkowski, and Hamming distances and among other. The hyperparameter plot is essential for selecting the k-value and distance metric, thereby minimizing error metrics or maximizing the coefficient of determination. Figure 7b indicates that most data points, associated with a lower objective function, fall at the lower end of the k-value spectrum. This suggests that while various distance metrics are suitable, optimal accuracy requires lower k-values.

Figure 7c summarizes the variance for both the training/validation and testing associated with the selection of Euclidean distance. The results indicate a systematic decrease in variance with increasing k-values, suggesting that a lower k-value, typically k = 1, significantly minimizes the disparity between the numerically computed model and the experimental TGA data of the pulverized DPB biomass material. This assertion is further corroborated by the increasing estimated uncertainty with rising k-values, as depicted in Figure 7d. Additionally, Table 5 presents statistical measures of dispersion for the training/validation and testing datasets, demonstrating decreasing skewness and kurtosis, which approach a normal distribution, with a systematic reduction in k-value. With a k-value of 1, the estimated coefficients of determination for the training/validation and testing datasets are 99.8% and 97.5%, respectively, which is regarded as an excellent level of performance in this study and serves as a strong indicator of the KNN modeling efficacy on the 20% unseen data.

**Figure 8:**
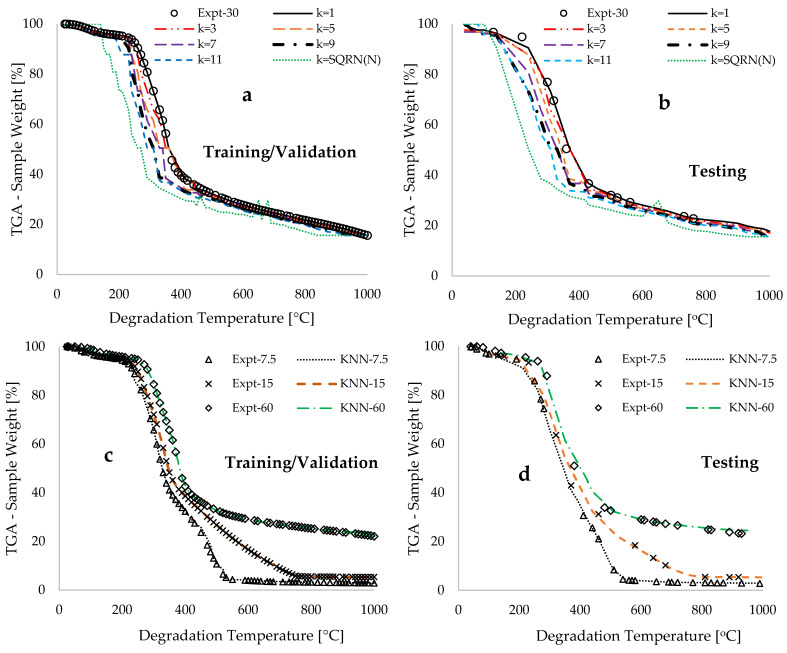
Graphical representations of the experimentally measured and k-nearest neighbors (KNN) predicted thermogravimetric analysis (TGA) sample weight percentages against temperature: (**a**) training and validation data at 30 °C·min^−1^ with varied k-values ranging from 1 to 23; (**b**) testing data at 30 °C·min^−1^ with varied k-values ranging from 1 to 23; (**c**) training and validation data for varied heating rates (7.5 to 60 °C·min^−1^) with a constant k-value of 1; and (**d**) testing data for varied heating rates (7.5 to 60 °C·min^−1^) with a constant k-value of 1.

Figure 8a,b present experimentally measured data points and KNN predicted TGA traces for varying k-values against degradation temperature at a constant heating rate of 30 °C·min^−1^, corresponding to both the training/validation and testing datasets. This shows a steady increase in the modeling performance with decreasing k-value, as previously discussed. This finding further supports the assertion in [85] by providing evidence that increasing k-values introduces significant bias into the model. This bias smooths out important local thermal characteristics, such as sharp transitions and local patterns of biomass degradation, which leads to under-fitting. Figure 8c,d show strong visual alignment and low error between experimental and KNN-predicted TGA traces (for k = 1) across various heating rates (7.5–60 °C·min^−1^) for both training/validation and testing datasets. A sensitivity analysis was conducted on the features to identify key parameters and their impact on the TGA traces obtained for the thermal devolatilization of the DPB biomass material. This approach is essential for optimizing key parameter(s), leading to enhanced efficiency in the biomass conversion or assisting in the design of reactors for such processes. The global sensitivity analysis (GSA) was implemented by exploring the entire range of the training/validation and testing datasets to understand their overall contribution to the model’s output uncertainty [86]. Typically, a ±20% variation in heating rates resulted in an estimated coefficient of determination of 98.53% when compared with the original data, while similar variations in time and degradation temperature yielded values of 99.45% and 97.53%, respectively. This suggests that the degradation temperature for the thermal devolatilization of DPB biomass is the most sensitive feature that could potentially alter the resulting TGA data, followed by heating rate and time. The estimated TGA profiles and thermokinetic data in this study demonstrate that temperature significantly influences the cleavage of chemical bonds, the release of volatile matter (hemicellulose and cellulose compositions), and the gradual degradation of lignin. The sensitivity analysis indicates that altering this feature, particularly at elevated temperatures, substantially changes the reaction types and decomposition profiles.

In comparison with the existing literature, the available research data regarding the application of machine learning tools in the analysis of the thermal devolatilization of palm waste indicates a significant advancement in the domains of thermal analysis, bioenergy, and sustainable waste management and valorization. For example, Zaifullizan et al. [87] conducted a comparative analysis that involved the application of artificial neural networks (ANN), support vector machines (SVM), and decision tree (DT) algorithms to predict thermogravimetric analysis (TGA) and derivative thermogravimetric (DTG) traces obtained for the pyrolysis and co-pyrolysis of various oil palm biomass types: empty fruit bunches (EFB), oil palm shells (OPS), and a blend of the two materials (EFB+OPS). The study [86] reported correlations for the predicted TGA ($R^{2}\sim 0.99$) and DTG ($R^{2}=0.94\;to\;0.99$) data for the three machine learning models in comparison with experimental data. The high accuracy obtained for the TGA data can generally be attributed to its smoother characteristics, which are less sensitive to noise, whereas the DTG traces, derived from the TGA data, inherently amplify noise. In a related study by this group [19], deep neural networks were employed to predict the thermal decomposition of palm fronds and polypropylene biocomposites, resulting in a reported R-squared value of 0.992. Furthermore, Ahmad et al. [88] utilized multivariate adaptive regression splines (MARS)—a machine learning tool—to model the activation energy calculated from the thermal devolatilization of oil palm empty fruit bunch as a function of heating rate, temperature, and conversion. This approach resulted in a reasonable correlation (R-squared = 0.987, MAE = 2.71, and RMSE = 4.94) between the modeling and experimental data. Consequently, the strong correlation between the KNN predictions and experimental results in this study further reinforces the versatility of the regression and classification model, enabling the extraction of key information from TGA datasets while addressing the complexity and non-linearity of the TGA traces obtained during the pyrolysis operation of DPB biomass.

## 4. Techno-Economic Assessments (TEA)

The analysis of the composition and the estimated thermokinetic data in this study show the technical feasibility and potential profitability of using date palm biomass (DPB) to produce bioenergy through fast pyrolysis. The primary thermal decomposition of the material happens between 200 and 530 °C (primarily consisting of bio-oil, bio-gas, and biochar [89]) and can contribute to a country’s net revenue through a rapid return on investment. This section provides a techno-economic assessment (TEA), giving insight into the technical feasibility, economic analysis, and environmental impact, using substantiated information from the existing literature and the results of this study.

### 4.1. Technical Feasibility

Biomass conversion technologies for bioenergy production are primarily divided into two categories: thermochemical (combustion, gasification, and pyrolysis) and biochemical (using microorganisms and enzymes [90]) methods. While both combustion and gasification can generate substantial energy from biomass, their adoption in thermochemical conversion is limited by several drawbacks. Gasification operations face challenges such as high tar formation and technical complexities in maintenance, while the combustion route is hindered by the release of noxious gases into the environment. In contrast, pyrolysis was chosen for this study because the heat-induced process can produce valuable biogas, bio-oil, and biochar, while also having the ability to handle wet biomass and inherently lowering emissions [91].

The proximate analysis data presented in Table 1 reveal that the inherent low moisture content of 4.47% makes the DPB biomass a valuable feedstock for thermochemical conversion into bioenergy. While the high volatile matter content of 72.01% suggests that the material is rich in hemicellulose and cellulose—which primarily decomposes at 200–530 °C into condensable products—the relatively low fixed carbon content of 18.14% indicates that biochar would be a secondary product; its yield and quality might be improved through torrefaction [92]. The low ash content of 5.38% is indicative of high quality, with reduced alkali and alkaline earth metals [93]. This characteristic is crucial for obtaining a high-quality and cleaner bio-oil product. Furthermore, the absence of sulfur in the elemental composition of the DPB biomass material is a positive aspect, as it reduces undesirable corrosive effects and potential Sox emissions. The moderate carbon content of 46.52% for this material suggests that achieving high biochar carbon concentration and stability is contingent upon reaching a sufficient pyrolysis temperature. Additionally, the high oxygen content of 46.52% may result in incomplete thermochemical conversion if the pyrolysis temperature is too low. However, a significant amount of moisture is removed via dehydration at lower temperatures (typically below 200 °C) and during the release of oxygen-containing volatile gases like carbon monoxide (CO) and carbon dioxide (CO_2_).

Evidently, the TGA/DTG traces and resulting thermokinetic data obtained for this thermochemical conversion process are impacted by the material’s composition. The relatively low values of activation energies calculated via the model-free kinetic models suggest that the heat-induced process can proceed at a reasonable speed once the minimum required initiation temperature has been met [80]. The positive and moderate values of the activation enthalpy and Gibbs free energy of the activation complex also suggest an endergonic, reactant-favored process that requires an external energy input for the formation of the more ordered activation complex (estimated negative entropy of action). In summary, both compositional and thermokinetic data suggest that the DPB biomass material is well-suited as a valuable pyrolysis feedstock leading to the formation of the main triplet products: bio-oil, bio-gas, and biochar.

### 4.2. Economic Analysis and Environmental Impact

The economic viability of fast pyrolysis of date palm waste has been reported [89] to be contingent primarily upon the availability and acquisition of feedstock, and the market prices for the product. The study [89] revealed that the economic viability of such thermochemical conversion plants situated in Saudi Arabia is viable and profitable due to the abundant and low purchasing cost of the feedstock. The possibility of producing triple products—bio-oil (upgraded to substitute fossil fuel-derived compounds), biogas (for thermal application and electricity generation), and biochar (for soil quality, water retention, and as an adsorbent for sequestering carbon)—represents a key indicator of a positive economic and environmental outlook. The study [89] reported that the Kingdom of Saudi Arabia could improve its gross domestic earnings (GDP) by USD 44.77 million per annum if nearly 50% of the total date palm waste is harnessed for bioenergy generation via fast pyrolysis. This scenario projects an internal rate of return (IRR) of 36.45% and a payback period of 2.57 years for a proposed 10 tons per day (TPD) bioenergy plant.

The date palm branches (DPB) material offers environmental advantages due to its absence of sulfur and low nitrogen composition [52], which results in minimal emissions of noxious gases. Pyrolysis of this material supports a sustainable waste management solution by diverting waste from landfills or the common practice of open burning, both of which cause environmental pollution. For a 10 TPD (tons per day) bioenergy plant, the potential reduction in carbon dioxide emissions from date palm waste pyrolysis amounts to 2000 tons annually [89], a significant contribution to the global goal of reducing atmospheric carbon footprint and promoting a circular economy model. Overall, the techno-economic assessment of the pyrolysis operation adopted in this study strongly supports the commercialization of bioenergy production from date palm branches/waste, offering both financial profitability and an environmentally friendly process.

### 4.3. The Necessity of Legal Frameworks

This study’s findings on the thermal decomposition of date palm waste demonstrate a potential pathway to transform agricultural waste into valuable products (biofuels). This transformation could support the technical requirements for establishing local bioenergy industries and align with Saudi Arabia’s Vision 2030 environmental objectives. Investigating the circular economy and legal framework for processing date palm waste into valuable products like bioenergy and biochar is essential for driving economic growth, supporting rural communities, and fostering environmental sustainability. Both Saudi Arabia and the UAE have created strong legal systems for waste management, using a mix of financial rewards and penalties to encourage waste recovery. The success of these frameworks, however, depends entirely on clear laws, strong enforcement by regulators, and private sector participation, which ultimately determines their impact on reducing waste and recovering resources. Government incentives, such as tax benefits, subsidies, and grants, encourage producers to proactively manage their waste by making sustainable practices financially viable and fostering innovation in waste processing technology. Regulatory clarity is urgently needed to establish consistent legal classifications for biochar and other date palm waste derivatives, thereby unlocking their significant economic and environmental benefits, and facilitating a transition to a sustainable, circular economy. Adopting circular production models, such as the circular economy and bioeconomy, is essential for effectively managing date palm waste in line with increasing global emphasis on these frameworks. This group is expected to undertake a comprehensive future study relating to the circular economy and legal framework concerning the utilization of date palm waste as an alternative bioenergy source both within Saudi Arabia and beyond. Such a study is expected to provide a framework for alternative uses, significantly reducing the carbon footprint through sustainable resource management.

## 5. Conclusions

This study provides the first comprehensive evaluation of the bioenergy potential of date palm branch (DPB) waste. The analysis specifically emphasizes reaction modeling, thermokinetic, the application of k-nearest neighbors (KNN) machine learning, and techno-economic assessments. Experimental analyses, including FTIR, SEM/XEDS, as well as elemental analysis of the pulverized date palm branch, elucidated its lignocellulosic characteristics and compositional attributes. TGA and DTG analyses of the DPB biomass, conducted between 25 and 1000 °C and heating rates between 7.5 and 60 °C·min^−1^, indicated that significant material degradation occurred between 200 and 530 °C, primarily attributed to the devolatilization of hemicellulose and cellulose components within the biomass. The application of Criado master plots to deconvoluted derivative thermogravimetric (DTG) traces, obtained using the least-squares method within the Gaussian mathematical functions, indicates that the R3 geometrical contracting cylinder model is the preferred reaction mechanism for the pseudo-components of moisture, hemicellulose, and cellulose. This is closely followed by the Jander [D3] and Ginstling–Brounshtein [D4] diffusion models. The estimated activation energies, calculated using the FWO, KAS, STK, and FR model-free kinetic methods, are 103.06 kJ·mol^−1^, 98.43 kJ·mol^−1^, 98.82 kJ·mol^−1^, and 109.30 kJ·mol^−1^, respectively. The estimated thermodynamic data in this study suggests that the formation of activated complexes (transition state) from the reactants is endothermic, endergonic, and indicative of a more structured state.

The implementation of the machine learning k-nearest neighbors (KNN) algorithm for the prediction of thermogravimetric analysis (TGA) traces proved to be successful. This was executed using an 80/20 data splitting strategy, alongside hyperparameter optimization that necessitated the selection of a k-value of 1, 30 epochs, and the use of Euclidean distance. This approach systematically reduced the training loss towards a local minimum. The trained KNN model achieved coefficients of determination of 99.8% and 97.5% for the 80% training/validation datasets and the 20% testing datasets, respectively, resulting in complete overlap and minimal errors between measurements and predictions. Global sensitivity analysis of the trained model indicates that degradation temperature is the primary factor affecting the pyrolysis of the DPB biomass, followed by heating rate and degradation time. Furthermore, the techno-economic assessments (TEA) indicate that leveraging date palm branches for bioenergy through fast pyrolysis is a feasible, financially rewarding, and environmentally friendly process, thereby promoting the sustainable utilization of this material within a circular economy model.

The research seeks to contribute to the Vision 2030 initiative of the Kingdom of Saudi Arabia (KSA) and the United Nations SDG 7 regarding energy transition and environmental sustainability (affordable and clean energy). This study demonstrates that the thermal devolatilization of date palm biomass (DPB) material offers significant advantages in sustainable waste management and bioenergy generation. The research provides valuable thermokinetic insights and, for the first time, applies the k-nearest neighbor machine learning tool to describe the local thermal characteristics of the materials, using data from thermogravimetric measurements. The study highlights the techno-economic assessment for the feasibility of bioenergy generation from such waste and further emphasizes that examining the legal framework for date palm waste valorization within a circular economy is a crucial area for future research.

## Figures and Tables

**Table 1:** Proximate and ultimate Analysis.

Proximate Analysis				Ultimate Analysis				
Moisture [%]	Fixed Carbon [%]	Volatile Matter [%]	Ash [%]	C [%]	H [%]	N [%]	S [%]	O [%]
4.47	18.14	72.01	5.38	45.54	5.40	2.54	0.00	46.52

**Table 2:** XEDS data for raw DPB and thermally decomposed biochar at 600 °C.

Element	Raw [wt%]	Biochar [wt%]
C	45.62 ± 0.92	4.46 ± 0.20
O	26.80 ± 1.24	35.16 ± 0.79
Al	0.31 ± 0.05	1.00 ± 0.07
Mg	0.62 ± 0.04	4.62 ± 0.15
Si	6.81 ± 0.33	23.80 ± 0.31
S	0.39 ± 0.03	0.85+ 0.05
Cl	2.53 ± 0.25	0.88 ± 0.07
K	2.79 ± 0.29	7.66 ± 0.22
Ca	4.02 ± 0.34	20.21 ± 0.39
Fe	0.89 ± 0.20	0.86 ± 0.18
Nb	0.67 ± 0.15	0.50 ± 0.05
Au	8.55 ± 0.63	0.00 ± 0.00
**Total**	**100**	**100**

**Table 3:** Statistical analysis of the estimated activated energy data.

	FWO	KAS	STK	FR
Mean Value (MV) [kJ.mol^−1^]	103.06	98.43	98.82	109.30
Median Value (MEDV) [kJ.mol^−1^]	114.12	110.32	110.71	119.17
Estimate uncertainty (Δ)	8.01	8.58	8.57	14.21
Range [kJ.mol^−1^]	22.65	24.25	24.24	40.18
Skewness (SKN)	−1.47	−1.62	−1.61	−2.29
Kurtosis (KTS)	1.36	2.10	2.07	5.64

**Table 4:** Average thermokinetic parameters for conversions ranging from 5 to 70%.

	R^2^ [-]	E_A_ [kJ.mol^−1^]	A [sec^−1^]	ΔS [kJ.mol^−1^.K^−1^]	ΔH [kJ.mol^−1^]	ΔG [kJ.mol^−1^]	*k* [-]
FWO	0.99	103.06	3.53 × 10^7^	−0.13	97.79	180.47	1.36 × 10^−15^
KAS	0.99	98.43	1.30 × 10^7^	−0.14	93.15	183.48	7.70 × 10^−16^
STK	0.99	98.82	1.24 × 10^−1^	−0.29	93.55	279.06	1.03 × 10^−23^
FR	0.94	109.30	3.92 × 10^8^	−0.13	104.03	189.69	2.37 × 10^−16^

**Table 5:** Estimate measure of dispersion of training and testing data.

	Mean	Median	Standard Deviation	Variance	Range	Skewness	Kurtosis	Uncertainty
Real train	44.651	29.651	34.862	1215.352	97.186	0.534	1.700	1.752
Real test	49.692	33.946	34.830	1213.155	97.142	0.362	1.530	3.501
Predict train k = 1	44.651	29.651	34.862	1215.352	97.186	0.534	1.700	1.752
Predict test k = 1	44.686	30.528	33.955	1152.953	97.186	0.582	1.792	3.413
Predict train k = 3	43.698	29.306	34.519	1191.540	97.186	0.577	1.763	1.752
Predict test k = 3	43.430	29.898	33.557	1126.067	97.186	0.638	1.879	3.413
Predict train k = 5	42.685	28.670	34.176	1168.014	97.186	0.622	1.832	1.717
Predict test k = 5	42.255	29.596	33.082	1094.387	97.186	0.687	1.970	3.325
Predict train k = 7	41.582	28.722	33.701	1135.746	97.186	0.679	1.941	1.694
Predict test k = 7	41.153	29.596	32.751	1072.614	97.186	0.749	2.088	3.292
Predict train k = 9	40.556	28.517	33.295	1108.548	97.186	0.679	1.941	1.694
Predict test k = 9	39.903	28.578	32.354	1046.808	97.186	0.749	2.088	3.292
Predict train k = 11	39.564	27.388	32.882	1081.232	97.186	0.781	2.137	1.652
Predict test k = 11	38.881	28.137	31.856	1014.802	97.186	0.856	2.332	3.202
Predict train k = SQRT(N)	36.162	25.769	31.516	993.235	97.186	1.033	2.707	1.584
Predict test k = SQRT(N)	35.293	26.981	30.767	946.588	97.186	1.100	2.912	3.092

## Data Availability

The original contributions presented in this study are included in the article/supplementary material. Further inquiries can be directed to the corresponding author.

## Supplementary Materials

The following supporting information can be downloaded at: https://www.mdpi.com/article/10.3390/polym17233182/s1, Table S1: Selected solid state reaction mechanisms (Adapted from [4]).
