# Supplementary material for: The Bioenergy Potential of Date Palm Branch/Waste Through Reaction Modeling, Thermokinetic Data, Machine Learning KNN Analysis, and Techno-Economic Assessments (TEA)
Source: Polymers (Basel). 2025 Nov 29;17(23):3182. doi: 10.3390/polym17233182 (PMC12694460; doi:10.3390/polym17233182)
Supplement: Supplementary file 1 [file polymers-17-03182-s001.zip › polymers-3955392-supplementary.pdf]

Supplementary Information

$$\frac{Z(x_i)}{Z(0.5)} = \frac{f(x_i).g(x_i)}{f(0.5).g(0.5)} = \left(\frac{T_x}{T_{0.5}}\right)^2 \frac{(dx/dt)_x}{(dx/dt)_{0.5}} \quad (S1)$$

where 0.5 represents conversion at x = 0.5.

Table S1. Selected solid state reaction mechanisms (Adapted from [4]).

Reaction Mechanisms	$g(x)$	$f(x)$
**Diffusion (DF) models**		
1D Diffusion model [D1]	x_i^2	$1/(2x_i)$
2D Diffusion model [D2]	$[(1-x_i).\ln(1-x_i)]+x_i$	$[-\ln(1-x_i)]^{-1}$
3D Diffusion model (Jander [D3])	$[1-(1-x_i)^{1/3}]^2$	$(3/2).(1-x_i)^{2/3}/[(1-(1-x_i)^{1/3})]$
Ginstling-Brounshtein [D4]	$1-(2x_i/3)-(1-x_i)^{2/3}$	$(3/2)/[((1-x_i)^{-1/3}-1)]$
Zhuravlev, Lesokin, Tempelman [D5]	$[(1-x_i)^{-1/3}-1]^2$	$(3/2).(1-x_i)^{4/3}/[((1-x_i)^{-1/3}-1)]$
Anti-Jander [D6]	$[(1+x_i)^{1/3}-1]^2$	$(3/2).(1+x_i)^{2/3}/[((1+x_i)^{1/3}-1)]$
**Geometrical contraction (GC) models**		
One-dimension [R1]	x_i	1
Contracting sphere [R2]	$1-(1-x_i)^{1/2}$	$(2).(1-x_i)^{1/2}$
Contracting cylinder [R3]	$1-(1-x_i)^{1/3}$	$(3).(1-x_i)^{2/3}$
**Reaction-order (RO) models**	$-\ln(1-x_i)$	$(1-x_i)$
First-order reaction model [F1]	$-\ln(1-x_i)$	$(1-x_i)$
Second-order reaction model [F2]	$(1-x_i)^{-1}-1$	$(1-x_i)^2$
Third-order reaction model [F3]	$[(1-x_i)^{-2}-1]/2$	$(1-x_i)^3$
One-third order [F1/3]	$1-(1-x_i)^{2/3}$	$(3/2).(1-x_i)^{1/3}$
Three-quarters order [F3/4]	$1-(1-x_i)^{1/4}$	$(4).(1-x_i)^{4/3}$
One and a half order [F3/2]	$(1-x_i)^{-1/2}-1$	$(2).(1-x_i)^{4/2}$
**Sigmoidal rate (SR) equations**		
Avarami-Erofe've [A3/2]	$[-\ln(1-x_i)]^{3/2}$	$(3/2).(1-x_i).[-\ln(1-x_i)]^{1/3}$
Avarami-Erofe've [A2]	$[-\ln(1-x_i)]^{1/2}$	$(2).(1-x_i).[-\ln(1-x_i)]^{1/2}$
Avarami-Erofe've (A3)	$[-\ln(1-x_i)]^{1/3}$	$(3).(1-x_i).[-\ln(1-x_i)]^{2/3}$
Avarami-Erofe've [A4]	$[-\ln(1-x_i)]^{1/4}$	$(4).(1-x_i).[-\ln(1-x_i)]^{3/4}$
Prout-Tomkins [Au]	$(x_i).(1-x_i)$	$\ln[x_i/(1-x_i)]$

Flynn-Wall-Ozawa (FWO): $ln\,[Q_R] = ln\left(\frac{A.E_A}{R.(g(x_i))}\right) - 5.331 - 1.052\left(\frac{E_A}{R}\right).\frac{1}{T}$ (S2)

Kissinger-Akahira-Sunose (KAS): $ln\,[Q_R/T^2] = ln\left(\frac{A.R}{E_A.(g(x_i))}\right) - \left(\frac{E_A}{R}\right).\frac{1}{T}$ (S3)

Starink (STK): $ln\,[Q_R/T^{1.92}] = ln\left(\frac{A.E_A}{R.(g(x_i))}\right) - \left(\frac{E_A}{R}\right).\frac{1}{T}$ (S4)

Friedmann Method (FR) $ln\left[Q_R\frac{dx_i}{dT}\right] = -\left(\frac{E_A}{R}\right).\frac{1}{T} + lnA + lnf(x_i)$ (S5)

Thermodynamic Parameters

Activation enthalpy: $\Delta H = E_A - R.T_M$ (S6)

Entropy of activation: $\Delta S = R.\ln\left(\frac{A.h}{K_B.T_M}\right)$ (S7)

Gibbs free energy of the activated complex: $\Delta G = \Delta H - T_M.\Delta S$ (S8)

Equilibrium constant: $k = \exp\,(-\Delta G/(E_A - \Delta H))$ (S9)

The symbol Q_R represents the heating rate, T designates the temperature, R is the gas constant, T_M denotes the maximum temperature of constant conversion, $g(x_i)$ and $f(x_i)$ represent the reaction mechanism functions, h denotes Planck's constant (6.626 x 10^{-34} J.s^{-1}), K_B signifies the Boltzmann constant (1.3806 x 10^{-23} J.K^{-1}), (ΔH is the activation enthalpy, ΔS is the entropy of activation , ΔG denotes the Gibbs free energy of the activated complex, and k the equilibrium constant.
